# Does Good Nebulization Therapy in the Emergency Room Reduce the Need for Hospitalization in Asthmatic Children?

**DOI:** 10.7759/cureus.41270

**Published:** 2023-07-02

**Authors:** Abdulhadi H Almazroea, Ahmad H Alharbi, Bushra A Alawfi, Bushra Q Alsaedi, Razan S Samman, Maryam A Almohalwas

**Affiliations:** 1 Pediatrics, Taibah University, Al-Madinah Al-Munawwarah, SAU; 2 Pediatric Emergency Medicine, Maternity and Children Hospital in Madinah, Al-Madinah Al-Munawwarah, SAU

**Keywords:** oxygen flow rate, position, hospitalization, nebulization therapy, asthma exacerbation

## Abstract

Introduction: Asthma is a chronic inflammatory disorder characterized by obstruction, hyperresponsiveness, and inflammatory changes in the airways. The overall prevalence of asthma in Saudi Arabian children ranges from 8% to 25%. Studies have shown that children who did not respond adequately to treatment in the emergency room (ER) were admitted to hospital for additional treatment which increased the cost and risk of hospital-acquired infections. The quality of nebulization therapy is influenced by several factors such as the position, dose, oxygen flow rate, and duration of treatment.

Objectives: In this study, we aimed to explore factors that affect nebulization therapy in ER and to assess the relation between nebulization technique in ER and hospital admission for asthmatic children, and these aims were achieved over the period from December 2021 to May 2023.

Methodology: An observational cross-sectional study was conducted in Maternity and Children Hospital (MCH) in Medina at the ER over the period from December 2021 to May 2023 for all children admitted to ER with asthma exacerbation. The sample size used to include patients in the study is 289 calculated using the Openepi website. Data were collected by observation and using medical records of the patients and analyzed using Statistical Package for Social Sciences (SPSS) version 26.0 (IBM Corp., Armonk, NY, USA).

Results: The total number of the sample was 289 children ages between two to 14 years. Sixty-four percent (n=185) reported as their gender as male while 36% (n = 104) as female. The median age of the children was four years old (interquartile range [IQR] = 4), and their median weight was 15 kg (IQR = 8.15). Also, more than 83% of the patients has mild asthma, while 16.3% of the sample were diagnosed with moderate to severe asthma. Besides, 92.4% of the sample was discharged from a hospital, and 76.5% received an appropriate dose of nebulization.

Discussion: After reviewing the results of the statistical analysis, the main findings were that the severity of asthma exacerbation was the most important factor influencing the outcome. It was found that 0.4% of patients with mild asthma were admitted to the hospital, compared to 44.7% of patients with moderate to severe asthma.

Conclusion: Our study assessed whether effective nebulization therapy in the ER will reduce the need for hospitalization in asthmatic children and the results indicate that the severity of asthma exacerbation was the most significant factor impacting hospital admission in asthmatic patients and influenced other factors of nebulization therapy. However, the other factors, such as the patient position, oxygen flow rate, and the dose of medications did not show any clinically significant impact on hospitalization rates.

## Introduction

Asthma is a chronic inflammatory disorder characterized by obstruction of the airways, hyperresponsiveness of the bronchial tubes, and underlying inflammatory changes. Three hundred million people worldwide are estimated to suffer from asthma [1]. According to studies conducted over the past three decades, asthma is one of the most common diseases in Saudi Arabia [2]. The overall prevalence of asthma in Saudi Arabian children ranges from 8% to 25% [3]. Bronchial asthma is a major public health problem that negatively impacts patients, their families, and the community by causing school loss, poor quality of life, frequent emergency visits, and hospitalizations [4].

Asthma exacerbation is a potentially life-threatening event, that is associated with increased healthcare use and decreased quality of life. There is an increase in the number of children with asthma exacerbations that require intensive care treatment and monitoring. Studies have shown that children who did not respond adequately to treatment in the emergency room (ER) were admitted for additional treatment until their symptoms subsided [5].

Generally, the first-line treatment for these children includes intermittent nebulized short-acting beta-agonists (SABA) every one to four hours, systemic corticosteroids (eg, oral prednisolone 1-2 mg/kg/day), and oxygen supplementation to reach oxygen saturation (SpO2) >95%. According to current clinical guidelines and experts, nebulized ipratropium bromide plus SABA is an effective adjunctive treatment [5]. Management of acute asthma exacerbation in the ER should target the following goals to achieve rapid reversal of bronchospasm, correction of hypoxemia if present, reducing the need for hospitalization, and preventing recurrence of the attack after discharge [3].

For the choice of inhalation system, the nebulizer is simple to use and it has the additional benefit of concomitant oxygen delivery and is therefore still the system of choice in treatment of acute severe asthma in all age groups [3]. Nebulizers are devices that aerosolize drugs so that they can be inhaled directly into the lower respiratory tract. They convert solutions into aerosols, which allows for easier inhalation [6]. The quality of nebulization therapy is influenced by several factors such as the position, dose, oxygen flow rate, and duration of treatment [7].

Good and effective nebulization therapy according to The Saudi Initiative for Asthma (SINA) guideline includes dose and duration of nebulization therapy for asthma exacerbation in children based on weight. Treatment of all grades of asthma exacerbation is by salbutamol for those who weighed less than 20 kg is 2.5 mg, and for those who weighed 20 kg or more, it ranges from 3.75 mg to 5 mg. Ipratropium bromide may be considered at a dose of 250 mcg by nebulizer every 20 minutes for the first hour only [3]. In addition, the positions have an impact on respiratory system mechanics. Supine position or lateral position does not seem beneficial for critically ill patients in terms of respiratory mechanics. The sitting position (with thorax angulation >30° from the horizontal plane) is associated with improvement of functional residual capacity (FRC), oxygenation and reduction of work of breathing [8]. Furthermore, the administration of oxygen to maintain saturation of at least 94% is recommended in all patients presenting with a moderate to severe asthma exacerbation [9]. Moreover, the oxygen should be at a rate of 6-8L/min [10].

In some of the previous studies, one meta-analysis confirmed the advantages of systemic corticosteroid administration within 60 minutes of arrival to ER in reducing hospital admissions [11]. Likewise, there is an observational study conducted in Thailand concluded that children who had insufficient nebulization treatment in the ER were more likely to be hospitalized. Thus, inadequate ER management may be considered a key factor influencing hospital admissions [5]. As far as the authors are aware, there are currently no known studies in Saudi Arabia that have evaluated the relationship between good nebulization therapy with the four factors, which are the position, dose, oxygen flow rate, plus duration of treatment and the hospital admission in children age group. Asthma exacerbation is a significant contributor to health care utilization as it can result in increased rates of hospitalization, usage of intensive care unit beds and the consumption of medical equipment and resources. Exacerbations of asthma result in significantly higher healthcare expenses for patients, as one study encountered in Saudi Arabia shows that the average in-patient medication expenditure is $1,976 [12]. As per the 2030 vision of Saudi Arabia and National Transformation Program 2020 vision, the Ministry of Health (MOH) has initiated multiple projects to ensure the cost-effective use of healthcare facilities [13]. For this sake, recognizing these predictors could reduce hospital admission rates and the significant financial burden to patients, families and society.

## Materials and methods

This prospective observational cross-sectional study was conducted in Maternity and Children Hospital (MCH) in Medinah at the ER over the period from December 2021 to May 2023 for all children admitted to ER with asthma exacerbation as the final diagnosis. The ethical committee of King Salman bin Abdulaziz Medical City Institutional Review Board granted approval for this study with the reference IRB number 032-22. Sample size used to include patients in the study was 289 as calculated using the Openepi website, assuming the following: anticipated percentage frequency is 25%, the confidence level is 95%, and the confidence limit was +/- 5.

Inclusion criteria included children aged between two to 14 years, patients who were diagnosed with an asthma exacerbation by the healthcare physician in the ER, as well as those who are identified as having a known history of bronchial asthma based on their medical records. In addition, our study includes patients with any grade of acute asthma exacerbation. For descriptive purposes, the sample was divided according to the Pediatric Respiratory Assessment Measure (PRAM) score, with the following components, suprasternal retraction, scalene muscle contraction, air entry, wheezing, and oxygen saturation, in which points between 0-3 (Mild), 4-7 (Moderate) and 8-12 (Severe) are given [14,15]. The weight was documented to calculate the optimal dose of medication for each patient. Exclusion criteria excluded patients with other comorbidities other than bronchial asthma.

An informed consent was signed by the participants or legal guardian at the time of recruitment. For all the patients suspected from the triage to have symptoms of acute asthma exacerbation, the research team followed them to the three areas, green, yellow, and red until the confirmation of the diagnosis was made by the ER physician. The patient position, oxygen flow rate and some of the PRAM score items were obtained by observation. Chest auscultation was done to complete the calculation of the PRAM score. In addition, the name of the drug used, dose, number of doses, duration of treatment and outcome, either hospitalization or discharge, were verified by reviewing the medical record of each patient.

To organize the data collection, collected information was written in a structured schedule and entered into an Excel file at the end of every week then analyzed by using Statistical Package for Social Sciences (SPSS; IBM Corp., Armonk, NY, USA). To ensure the confidentiality of patient data, it was stored securely in a file protected by a password that only the research team could access.

## Results

Statistical analysis

SPSS was used for data analysis. Data normality was tested using the Kolmogorov-Smirnov test. Continuous data were presented as the median and interquartile range (IQR), while categorical variables were represented as frequency and percentages. Categorical variable analysis was performed using Fisher’s exact test was used to determine if there was a significant association between the rate of admission to the hospital for patients` asthma and the factors that affect nebulization treatment. A p-value less than 0.05 was considered statistically significant, and the confidence interval was 95%.

Results

The total number of the sample was 289 children ages between two to 14 years. Table 1 illustrates the demographic characteristics of the sample, 64% (n=185) reported their gender as male while 36% (n = 104) as female. The median age of the children was four years old (IQR = 4), and their median weight was 15 kg (IQR = 8.15). Also, more than 83% of the patients has mild asthma, while 16.3% of the sample were diagnosed with moderate to severe asthma. Besides, 92.4% of the sample was discharged from a hospital, and 76.5% received an appropriate dose of nebulization.

**Table 1:** Demographic characteristics of participants (N=289) IQR: interquartile range

Characteristics	frequency	percentages
Age median (IQR) 4 (4)		
Weight median (IQR) 15 (8.15)		
Gender		
Male	185	64%
Female	104	36%
Pediatric Respiratory Assessment Measure- PRAM		
Patients with mild asthma	242	83.7%
Patients with moderate to severe asthma	47	16.3%
Outcome		
Discharge	267	92.4%
Admission	22	7.6%
Dose to weight		
Appropriate	221	76.5%
Low dose	66	22.8%
High dose	2	0.7%
O_2_ flow rate		
O_2 _< 6L	235	81.3%
6L - 8L	45	15.6%
O_2_ > 8	9	3.1%
Position		
45 degrees	24	8.3%
Sitting	257	88.9%
Supine	8	2.8%

After reviewing the results of statistical analysis, severity of asthma exacerbation was the most important factor influencing the outcome. It was found that 0.4% of patients who had mild asthma were admitted to the hospital, compared to 44.7% of patients who had moderate to severe asthma. Regarding dose-weight appropriateness, out of 289 patients, 221 (76.5%) received appropriate dose of medication. On the other hand, 66 (22.8%) patients were given low dose and two patients only, accounting for 0.7% of the whole sample, received high dose of treatment (Table 1).

From the point of view of O2 flow rate, the sample was divided into three categories. Forty-five patients (15.6%) received the optimal rate of O2 delivery which was 6-8L/min, while 235 (81.3%) patients which represented the majority of the sample were given O2 flow rate less than 6L/min, and a minority of only nine patients (3.1%) received O2 at a rate more than 8L/min. The position was also investigated. The number of patients receiving treatment while in a sitting position was 257 (88.9%) and 24 patients (8.3%) were at 45°. The two previously mentioned positions are considered optimal positions for nebulization. Only eight patients (2.8%) were in a supine position, which is the least favorable position for nebulization (Table 1).

**Figure 1:**
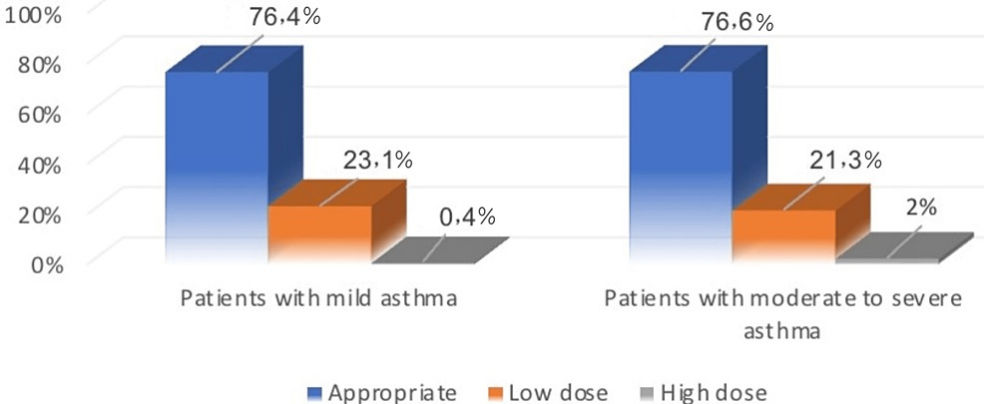
Difference between patients with mild asthma and moderate to severe asthma on the dose of treatment

Figure 1 illustrates that 76.4% of patients who had mild asthma received an appropriate dose of nebulization, while 23.1% of them received a low dose. Also, 76.6% of patients who had moderate to severe asthma received an appropriate dose of nebulization, while 21.3% of them received a low dose.

**Figure 2:**
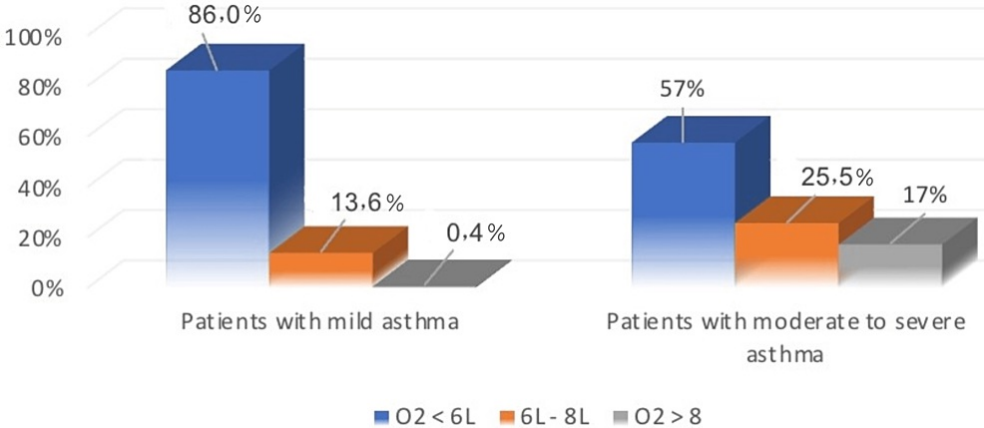
Difference between patients with mild asthma and moderate to severe asthma on O2 flow rate.

Figure 2 shows that 86% of patients who had mild asthma received a rate less than 6L, while 13.6% of them received between 6-8L of O2. Also, 57% of patients who had moderate to severe asthma received less than 6L of O2, while 25.5% of them received between 6-8L of O2.

**Figure 3:**
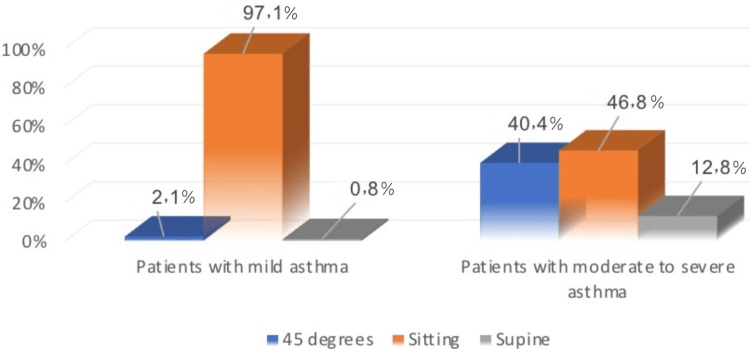
Difference between patients with mild asthma and moderate to severe asthma on patient position

Figure 3 illustrates the position of patients when they received treatment; 97.1% of patients who had mild asthma were sitting, while 2.1% of them were in a 45° position. Also, 46.8% of patients who had moderate to severe asthma were sitting, while 40.4% of them were in a 45° position when they received treatment.

**Figure 4:**
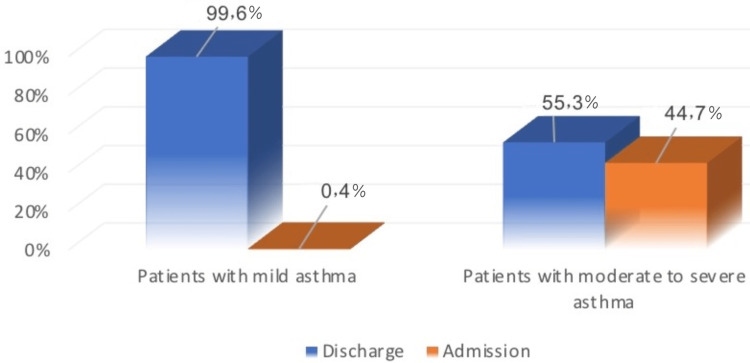
Difference between patients with mild asthma and moderate to severe asthma on the rate of admission to hospital.

Figure 4 illustrates that 0.4% of patients who had mild asthma were admitted to the hospital, compared to 44.7% of patients who had moderate to severe asthma.

In Table 2, Fisher's exact test shows there was a statistically significant association between admission or non-admission to a hospital and O2 flow rate (P < .001). Post hoc comparisons revealed that higher rates of discharge from the hospital were seen among those patients who received less than or equal to 8L of oxygen flow rate. In comparison, patients who received more than 8L of oxygen flow rate registered higher rates of admission to the hospital. Also, there was a statistically significant association between admission or non-admission to a hospital and the patient's position when he received treatment (P < .001).

**Table 2:** Association between admission and non-admission to the hospital with factors affecting nebulization for asthmatic patients (N=289). * P-value < 0.05 is statistically significant.

Factors		Outcome		P-value
		Discharge	Admission	
Dose to weight	Appropriate	204 (70.6%)	17 (5.9%)	1.00
	Low dose	61 (21.1%)	5 (1.7%)	
	High dose	2 (0.7%)	0 (0%)	
O_2_ flow rate	O_2 _< 6L	227(78.5%) *	8 (2.8%)	< .001>
	6L - 8L	38 (13.1%) *	7 (2.4%)	
	O_2_ > 8	2 (0.7%)	7 (2.4%) *	
Position	45 degrees	10 (3,5%)	14 (4.8%) *	< .001*
	Sitting	254 (87.9%) *	3 (1%)	
	Supine	3 (1%)	5 (1.7%) *	
Nebulization technique	Optimal	2 (0.7%)	5 (1.7%) *	< .001*
	Other	265(91.7%) *	17 (5.9%)	

Post hoc comparisons revealed that higher rates of admission to the hospital were seen among those patients who were in 45° and supine positions. In comparison, patients who received treatment in a sitting position registered higher rates of discharge from the hospital. Finally, results show there was a statistically significant association between admission or non-admission to a hospital and nebulization technique (P < .001).

Post hoc comparisons revealed that higher rates of admission to the hospital were seen among those patients who received optimal treatment (appropriate dose, 6-8L O2 flow rate, and 45° position). In comparison, patients who received other levels of nebulization treatment registered higher rates of discharge from the hospital.

Table 3 illustrates the comparison of the rate of admission or non-admission to a hospital among patients with mild asthma. Fisher's exact test shows there was a statistically significant association between admission or non-admission to a hospital and O2 flow rate (P =.004). Post hoc comparisons revealed that higher rates of discharge from the hospital were seen among those patients who received less than 6L of oxygen flow rate.

**Table 3:** Association between admission and non-admission to the hospital with factors affecting nebulization for mild asthmatic patients (N=242) * P-value < 0.05 is statistically significant.

Factors		Outcome		P-value
		Discharge	Admission	
Dose to weight	Appropriate	185 (76.4%)	0 (0%)	.236
	Low dose	55 (22.7%)	1(0.4%)	
	High dose	1 (0.4%)	0 (0%)	
O_2_ flow rate	O_2 _< 6L	208 (86%) *	0 (0%)	.004*
	6L - 8L	33 (13.6%)	0 (0%)	
	O_2_ > 8	0 (0%)	1 (0.4%) *	
Position	45 degrees	4 (1.7%)	1 (0.4%)	.028*
	Sitting	235 (97.1%) *	0 (0%)	
	Supine	2 (0.8%)	0 (0%)	
Nebulization therapy	Optimal	0 (0%)	0 (0%)	-
	Other	241 (99.6%)	1 (0.4%)	

In comparison, patients who received more than 8L of oxygen flow rate registered higher rates of admission to the hospital. Also, there was a statistically significant association between admission or non-admission to a hospital and the patient's position when he received treatment (P = .028). Post hoc comparisons revealed that higher rates of discharge from the hospital were seen among those patients who received treatment in 45° and sitting positions.

Table 4 illustrates the comparison of the rate of admission or non-admission to a hospital among patients with moderate to severe asthma. Fisher's exact test shows there was a statistically significant association between admission or non-admission to a hospital and O2 flow rate (P =.041). Post hoc comparisons revealed that higher rates of discharge from the hospital were seen among those patients who received less than 6L of O2 flow rate.

**Table 4:** Association between admission and non-admission to the hospital with factors affecting nebulization for moderate to severe asthmatic patients (N=47) * P-value < 0.05 is statistically significant.

Factors		Outcome		P-value
		Discharge	Admission	
Dose to weight	Appropriate	19 (40.4%)	17 (36.2%)	.853
	Low dose	6 (12.8%)	4 (8.5%)	
	High dose	1 (2.1%)	0 (0%)	
O_2_ flow rate	O_2 _< 6L	19 (40.4%) *	8 (17%)	.041*
	6L - 8L	5 (10.6%)	7 (14.9%)	
	O_2_ > 8	2 (4.3%)	6 (12.8%)	
Position	45 degrees	6 (12.8%)	13 (27.7%) *	< .001>
	Sitting	19 (40.4%) *	3 (6.4%)	
	Supine	1 (2.1%)	5 (10.6%) *	
Nebulization technique	Optimal	2 (4.3%)	5 (10.6%)	.127
	Other	24 (51.1%)	16 (34%)	

Also, there was a statistically significant association between admission or non-admission to a hospital and the patient's position when they received treatment (P < .001). Post hoc comparisons revealed that higher rates of admission to the hospital were seen among those patients who were in 45° and supine positions. In comparison, patients who received treatment in a sitting position registered higher rates of discharge from the hospital.

## Discussion

This study highlighted the factors that affect nebulization therapy in the ER and assessed the relation between nebulization technique in ER and hospital admission for asthmatic children. The optimal treatment of asthma exacerbation included a sitting position (with thorax angulation >30° from the horizontal plane) which was considered as 45°. Medications as per disease conditions, and dose to be calculated as per weight.

Treatment of all grades of asthma exacerbation is by salbutamol for those who weighed less than 20 kg is 2.5 mg, and those who weighed 20 kg or more it ranges from 3.75 mg to 5 mg. Ipratropium bromide may be considered at a dose of 250 mcg by nebulizer every 20 min for the first hour only. Optimal oxygen flow rate should range between 6-8L/min. Other medications may be considered according to severity, such as; magnesium sulphate, oral/IV corticosteroids and inhaled epinephrine.

From a financial point of view, asthma-related emergency admissions were associated with a high cost for medication utilization. One of our expectations was that whenever the patient was exposed to the wrong nebulization technique there would be more hospital admissions, leading to high economic burden among patients. During the period of research conduction, we found that the average medical cost (pharmaceuticals) in ER is $108 for each patient, while the average cost for patients who are admitted in the ward is $945, and the estimated cost for the patients who are admitted in the ICU is $2,923. According to that point, it is mandatory to encourage emergency physicians to follow local guidelines to minimize hospitalization rates.

Our findings are consistent with a previous study conducted in Mecca, Saudi Arabia during the pilgrim season by Hasseb et al. in 2020. The study highlighted the impact of asthma-related admission at emergency facilities dealing with pilgrims during this season in which the mean age of patients was 50±1.03 and more than half of the patients were admitted with mild asthma exacerbations (61.9%) followed by moderate severity (37%). The total estimated medical cost was $9.87 with an average cost of $36.5 for each patient. The majority of patients (89.7%) were cured and discharged after receiving medical care in an emergency setting [13]. In contrast to the previous study, the mean age in our study was between two and 14 years, conducted in the whole year with no specific season. In addition, there were life-threatening cases that presented in 16.3% of the sample. Furthermore, the average medical cost in ER is $108 for each patient, which was higher than the comparison study.

Area of further work

Due to important limitations in study design, further prospective clinical studies are needed to confirm our findings before clinical implementation. Since most patients who are admitted are patients with moderate or severe asthma exacerbation, further studies with a large moderate and severe sample are needed to evaluate the response to the nebulization technique and its effects on rate of admission. Also, to make the results more accurate, it is better to equalize the sample number in all categories (Mild, Moderate, Severe) to overcome the severity effect on other factors.

Study strengths and limitations

A major strength of our study lies in its direct observation of oxygen flow rate, position and PRAM score. Other patient information such as medication, dose, weight and outcome were obtained and confirmed by reviewing medical records. As far as the research team knows, this study is the first of its kind in Saudi Arabia in evaluating the properness of nebulization technique by spotlighting the different factors affecting it and its relation with hospital admission, especially in the pediatric age group. The major limitation of this study was its prospective nature and that the patients included in the study were selected depending on their presence in the ER the day of data collection regardless of the severity of their asthma exacerbation and that may indirectly affect the generalizability of the results. Also, this study is a single-center study and probably will not reflect national asthma care and control as the level of care is diverse across the country. Moreover, there are not many studies discussing the factors affecting nebulization technique and further admission or discharge for asthmatic patients, which made it more difficult for the research team to evaluate the findings and compare to with other studies.

## Conclusions

In conclusion, our study assessed whether effective nebulization therapy in the ER will reduce the need for hospitalization in asthmatic children and the results indicate that the severity of asthma exacerbation was the most significant factor impacting hospital admission in asthmatic patients and influenced other factors of nebulization therapy. However, the other factors, such as the patient position, oxygen flow rate, and the dose of medications did not show any clinically significant impact on hospitalization rates.
